# The Innate and Adaptive Immune Functions of Osteoblast-Lineage Cells

**DOI:** 10.1007/s11914-026-00970-5

**Published:** 2026-05-12

**Authors:** Mohammad A. Hossain, Plinio R. Hurtado, Gerald J. Atkins

**Affiliations:** 1 Biomedical Orthopaedic Research Group, Discipline of Orthopaedics and Trauma, School of Medicine, Adelaide University, Adelaide, SA Australia; 2 Central and Northern Adelaide Renal and Transplantation Service, Royal Adelaide Hospital, Adelaide, South Australia Australia; 3 Discipline of Medicine, School of Medicine, Adelaide University, Adelaide, SA Australia

**Keywords:** Osteoblast, Osteocyte, Innate immunity, Adaptive immunity, Immune response, Osteomyelitis

## Abstract

**Purpose of the Review:**

The osteoblast lineage has traditionally been viewed through a structural and metabolic lens, yet growing evidence indicates that these cells possess diverse functions, including roles in innate and adaptive immune responses. To establish a coherent mechanistic framework, we performed a systematic review of the literature concerning immune signalling, antigen presentation, and pathogen responses across the osteoblast lineage.

**Recent Findings:**

We identified 463 unique studies, with 43 meeting the inclusion criteria. Our synthesis reveals a stage-specialised immune continuum. Osteoprogenitors appear to initiate early inflammatory signalling while mature osteoblasts operate as microbial sensors and conditional antigen-presenting cells via inducible expression of cell membrane and cytosolic pattern recognition receptors, and a functional major histocompatibility complex class II apparatus. Osteocytes, the most abundant and long-lived bone cell type, are also capable of detecting microbial danger and activate extensive interferon, chemokine and cytokine programmes within the lacunocanalicular network.

**Summary:**

Together, these properties define a stromal immune system that coordinates both innate and adaptive immunity across bone surfaces and the mineralised matrix. This osteoblast lineage-integrated immune architecture provides a conceptual basis for understanding osteomyelitis incidence and persistence, biomaterial–immune interactions, as well as inflammatory bone remodelling, reframing this lineage as a previously underappreciated regulator of skeletal and systemic immunity.

## Introduction

Bone has traditionally been viewed as a mechanically specialised organ maintained by the coordinated activities of osteoblasts, osteoclasts and osteocytes [1–4]. Within this framework, the osteoblast lineage, consisting of osteoprogenitor cells, pre-osteoblasts, mature osteoblasts, bone lining cells and osteocytes, has been largely defined by its metabolic roles in bone matrix synthesis and mineralisation, and mechanotransduction. Within the umbrella term osteoimmunology, multiple effects of immune cells, cytokines and chemokines on bone remodelling in health and disease, have been described, as well reviewed in detail elsewhere [5, 6]. These include regulation of the RANKL/OPG axis in osteoblastic cells, driving or inhibiting the differentiation and/or activation of bone resorbing osteoclasts, as well as regulating osteogenesis. In addition, a substantial body of evidence suggests that osteoblast lineage cells participate as a stroma in supporting haemopoiesis, including myelopoiesis, as well as B and T lymphopoiesis, at least to some extent in a bone remodelling stage-selective manner [7–9]. However, accumulating evidence from the fields of infection pathobiology, biomaterials research and stromal immunology suggests that the osteoblast lineage, with reports from both osteoblast and osteocyte-focussed studies, also participates directly in innate and adaptive immunity [11, 12].

At the 2025 International Consensus Meeting on Musculoskeletal Infection, it was recognised with strong consensus (98%) that persistent intracellular infection by a wide range of pathogens occurred throughout the osteoblast lineage and that these infections contributed to disease propagation and pathogenesis [13]. This is arguably best characterised in the context of periprosthetic joint infection (PJI). PJI is a serious complication following joint replacement surgery, occurring either perioperatively or later, most commonly via haematogenous seeding of the implanted hardware; pathogens forming biofilms on these surfaces can then spread to the surrounding osseus and non-osseus tissues [14, 15]. The presence of osteomyelitis is a recognised risk factor for chronicity and poor outcomes in PJI treatment [16, 17].

The question arises, to what extent do the innate and adaptive immune functions of the infected or pathogen-exposed osteoblasts and osteocytes contribute to, or indeed protect against, osteomyelitis? Reports across the past two decades describe osteoblast responsiveness to microbial and inflammatory cues, robust secretion of cytokines and chemokines, and production of antimicrobial peptides, characteristic of innate immune responsiveness [11, 18]. More unexpectedly, osteoblasts have been shown to express the adaptive immune response hallmark, major histocompatibility complex class II (MHC class II), as well as other key components of the antigen presentation machinery, raising the possibility that osteoblasts can acquire context-dependent antigen-presenting cell (APC) functions [11, 12]. Such findings suggest that osteoblasts may bridge pathogen sensing and T-cell activation at the bone–bone marrow interface, a concept that remains under-recognised and mechanistically unresolved.

Osteocytes, comprising approximately 90% of all bone cells and residing deep within the lacunocanalicular network, have been even more overlooked as potential immune contributors. Historically conceptualised as primarily mechanosensitive regulators of bone remodelling, osteocytes are now implicated in the control of physiological bone remodelling and inflammatory bone loss, in particular through the support of osteoclastogenesis via regulation of the RANKL/RANK/OPG axis [4, 19], regulation of bone matrix integrity and local and systemic mineral homeostasis through osteocytic osteolysis and fibroblast growth factor 23 (FGF23) expression, respectively, and are also implicated directly in the response to bacterial infection [4, 20, 21].

Despite these intriguing findings, the field lacks an integrated framework describing how innate and adaptive immune properties are distributed across the osteoblast lineage, whether these immune functions are conserved or specialised across stages of differentiation and how osteoblasts and osteocytes collectively influence local and systemic immunity. Past studies have been heterogeneous in model systems, pathogens, stimuli and readouts, resulting in mechanistic fragmentation and the absence of a coherent biological model. To resolve this gap, we performed a systematic review of the literature to synthesise current evidence on immune capabilities distributed across the osteoblastic lineage.

## Methodology

### Literature Search Strategy and Study Selection

A systematic literature search was conducted of two databases, PubMed and Embase, to identify studies investigating the immune functions of osteoblast-lineage cells. Searches were performed using predefined combinations of controlled vocabulary and free-text terms related to osteoblasts, osteocytes, immune responses and infection. The initial searches retrieved 552 abstracts from PubMed and 272 from Embase. Retrieved search results were uploaded into Covidence systematic review software (Covidence, Melbourne, VIC, Australia). After removal of duplicates, all remaining 463 abstracts were screened for relevance. Studies were eligible for inclusion if they investigated immune functions of osteoblast-lineage cells, specifically, either (1) professional immune functions, such as antigen presentation via MHC class II, co-stimulatory molecule expression or direct activation of immune cells, or (2) non-professional/innate immune functions, including cytokine and chemokine production, pathogen sensing, recruitment of immune cells and participation in inflammatory or infectious bone pathologies, such as osteomyelitis and PJI.

**Fig. 1:**
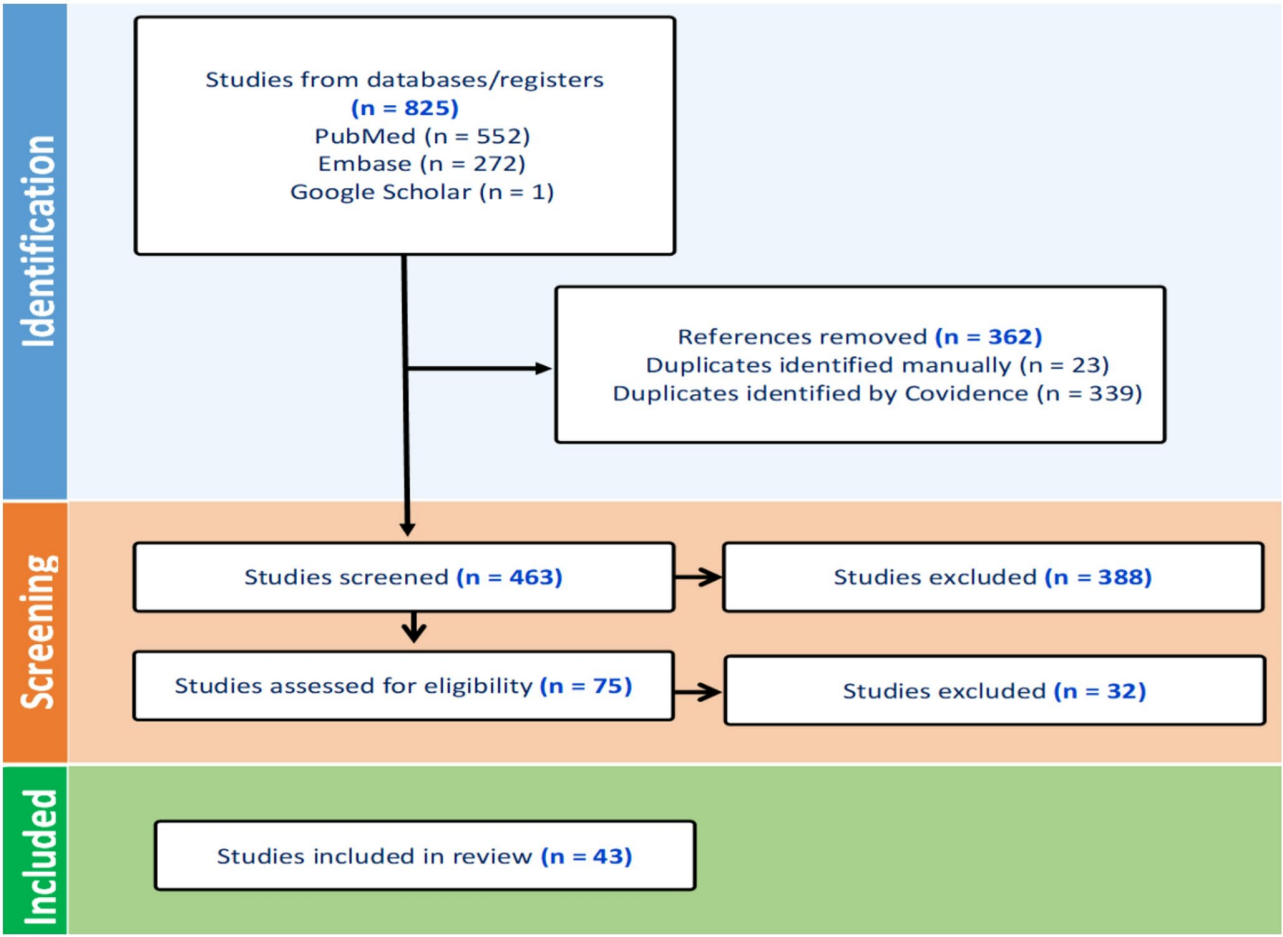
PRISMA flow diagram summarising study selection for the systematic review The diagram outlines identification, screening and eligibility assessment of records from PubMed, Embase and manual searches, resulting in 43 studies included in the final synthesis

Studies were excluded if they focused solely on bone remodelling, mineral density, fracture healing or other skeletal processes without an explicit immunological component, or if they did not investigate osteoblast-lineage cell immune-related responses. Following abstract screening, 75 articles were selected for full-text review. Of these, 43 studies met the final inclusion criteria and were included in the qualitative synthesis. The study selection process is summarised in Fig. 1.

## Results

### Overall Patterns Across the Osteoblast Lineage

Across the included studies, experimental systems encompassed mesenchymal stromal cell-derived osteoprogenitors, immortalised osteoblast-like cell lines, human and murine primary osteoblasts, and ex vivo or in situ analyses of osteocytes embedded within native bone tissue. Despite the substantial heterogeneity in cell models and stimuli tested, a coherent set of overarching patterns was evident, as summarised below.

### Innate Immune Sensing Across the Osteoblast Lineage

Multiple studies demonstrated that osteoblast-lineage cells express a functional repertoire of pattern-recognition receptors (PRRs), responsible for detection of a range of conserved microbial structures termed pathogen associated molecular patterns (PAMPs), which are fundamental to innate immunity [22]. These included Toll-like receptors (TLR), NOD-like receptors and, in selected models, nucleic acid-sensing pathways, such as the cyclic GMP-AMP synthase-stimulator of interferon gene (cGAS–STING) pathway (Table 1**)** [23–25]. In human and mouse primary osteoblasts, bacterial pathogens, including *Staphylococcus aureus*, *Mycobacterium tuberculosis* and *Chlamydia pneumoniae*, as well as purified PAMPs, such as lipopolysaccharide (LPS), lipopeptides, polyinosinic: polycytidylic acid (Poly I:C) and unmethylated CpG DNA motifs, consistently induced PRR-dependent transcriptional responses [24–26]. Studies using human and mouse osteoblastic cell lines (MG-63, Saos-2 and MC3T3-E1) recapitulated many of these responses, although with quantitative and qualitative variation across systems [25, 27–30].

**Table 1:** Evidence for innate immune sensing across the osteoblast lineage

Cell type	Key PRRs Identified	Stimuli/Pathogen	Downstream Signalling	Functional Immune Output
Human primary osteoblasts	TLR2, TLR4 [25, 65]	*S. aureus*, *P. gingivalis* *M. tuberculosis*, LPS [18, 37, 66]	NF-κB, IRF3, MAPK, Type 1 IFN [18, 65, 67]	IL-6, IL-12, CXCL8, CCL2, CXCL10, CCL2, β-defensins [18, 65, 66]
Mouse primary osteoblasts	TLR2, TLR4, TLR3, NOD2 [11, 23, 26, 68]	*S. aureus*, *S. enterica* [18, 26, 68] LPS-pulsed splenocytes [23]	Type 1 IFN [11, 26]	IL-6, IL-12, CCL2, CCL5, CXCL10, CXCL13, IFN-β [11, 18, 23, 26]
Human osteoblast cell line: MG-63, Saos-2	TLR2/4, NOD pathways [28]	bacterial lysates, TLR ligands [29]	NF-κB, JNK/p38 [36, 69]	IL-6, CXCL8, chemokines [28, 29, 36, 66, 68, 70, 71]
Mouse osteoblast cell line: MC3T3-E1	TLR2, TLR4 [28, 49]	*S. aureus*, TLR ligands [28]	NF-κB, GSK-3β/ Wnt/β-catenin [49]	LL-37, antimicrobial peptides (panel), IL-6, IL-1β, TNF [28, 30, 49] CXCL5 [30]
Human primary osteocytes (in situ, bone explant-derived)	TLR2, NOD2	*S. aureus* lacunocanalicular invasion [10]	ISGs, Type 1 IFN [10]	MMP1, MMP3, MMP13, CXCL1, CXCL8, CXCL9, CXCL10, CXCL11, CCL5, other inflammatory mediators [10, 20]
Human osteocyte cell line: SaOS2-OY	TLR2	*S. aureus*	Not reported	CXCL6, CXCL9, CXCL10, CCL5, MMP1, MMP13 [29]
Mouse osteocyte cell line: MLO-Y4	TLR2	*S. aureus*	COX	Antimicrobial peptides (panel), IL-6 [28]

*CCL* C–C motif chemokine ligand, *CXCL* C–X–C motif chemokine ligand, *COX* cyclooxygenase, *GSK-3β* glycogen synthase kinase-3 beta, *IFN* interferon, *IFN-β* interferon beta. *IL* interleukin, *IRF3* interferon regulatory factor 3, *ISG* interferon-stimulated gene, *JNK* c-Jun N-terminal kinase, *LPS* lipopolysaccharide, *LL* LPS binding protein-LPS, *M. tuberculosis* Mycobacterium tuberculosis, *MAPK* mitogen-activated protein kinase, *MMP* matrix metalloproteinase, *NF-κB* nuclear factor kappa-light-chain-enhancer of activated B cells, *NOD2* nucleotide-binding oligomerisation domain-containing protein 2, *P. gingivalis *Porphyromonas gingivalis, *PRR* pattern-recognition receptor, *PJI* periprosthetic joint infection, *S. aureus *Staphylococcus aureus, *S. enterica* Salmonella enterica, *TLR* Toll-like receptor, *TNF* tumour necrosis factor, *Wnt* wingless integration

Several studies demonstrated intracellular recognition of invasive or phagocytosed bacteria, with localisation of pathogens to endosomal–lysosomal compartments and activation of downstream NF-κB, IRF and MAPK signalling cascades [27, 28]. In systemic inflammation and sepsis models [31], osteoblasts in vivo exhibited PRR-linked transcriptional signatures, supporting the concept that circulating inflammatory danger signals are directly perceived at the bone-bone marrow interface.

Evidence for innate immune sensing in osteocytes was more limited but nevertheless convergent. In vitro and in vivo models of osteomyelitis and PJI, as well as analysis of infected bone explants, revealed upregulation of PRR-associated transcripts and interferon-stimulated genes in osteocytes within the lacunocanalicular network [20, 29]. In these contexts, bacterial penetration of canaliculi correlated with osteocyte activation patterns that are most parsimoniously explained by PRR engagement, even though direct receptor expression has not been exhaustively profiled [25, 28, 29]. One study comparing MC3T3-E1 osteoblast with MLO-Y4 osteocyte responses to *S. aureus* reported stronger antimicrobial peptide (LL-37) responses in osteoblasts and stronger proinflammatory (IL-6) responses in osteocytes, suggesting an inherent difference in response pattern based on microenvironmental localisation of the respective cell stage [28], with the limitation of inherent significant differences between these cell lines in terms of skeletal origin, genetic background and the immortalization methodology used in their derivation [32, 33].

### Divergent Cytokine and Chemokine Programs Along the Lineage

Across experimental systems, osteoblasts were reported to behave as competent secretory innate immune cells, mounting robust cytokine and chemokine responses following stimulation (Table 2). Proinflammatory cytokines, including IL-6, CXCL8, TNF and, in some contexts, type 1 interferons, were induced by PAMPs, such as live bacterial and viral mimetics known to promote immune cell recruitment, amplify inflammatory signalling, and enhance antigen presentation [18, 34]. In parallel, chemokines were consistently upregulated, including neutrophil-attracting chemokines (CXCL1, CXCL5, CXCL6, CXCL8), monocyte-recruiting chemokines (CCL2), and T cell/natural killer (NK) cell–associated chemokines (CXCL10, CCL5), highlighting the potential ability of osteoblasts to orchestrate coordinated recruitment of innate and adaptive immune cells to sites of bone infection or inflammation [23, 34–38].

**Table 2:** Cytokine and chemokine programs across osteoblast and osteocyte populations

Cell type	Dominant cytokine/chemokines	Inducing Stimuli	Functional Immune output
Human primary osteoblasts	IL-6, CXCL8, TNF, CCL2, CXCL10 [25, 35, 36, 46, 67]	*S. aureus*, *M. tuberculosis*, IL-1β, TNF, IL-17 [25, 35]	Neutrophil/monocyte recruitment [24, 36]
Mouse primary osteoblasts	IL-6, CXCL8, TNF, CCL2, CXCL10, IFN-β [11, 23, 45, 72, 73]	*S. aureus*, *M. tuberculosis*, IL-1β, TNF, LPS, IL-17 [26, 45, 73]	Innate immune activation; chemokine-driven leukocyte recruitment [11, 23, 45, 72–74]
Osteoblast lines (MG-63, Saos2, MC3T3-E1)	IL-6, CXCL8, CCL2, CXCL10 [27, 38, 75]	TLR ligands, bacterial lysates [49]	Mechanistic immune models [27, 38, 49]
Osteocytes (ex vivo / in situ)	ISGs, IL-6 (modest) [28]	Osteomyelitis, PJI [10, 20, 28]	Deep-bone inflammatory signalling [10, 20, 29]
Osteoprogenitors (Mouse ST-2 cell line)	IL-6, CXCL8, CCL2, CCL5, CXCL10, VEGF [25, 46, 76]	Inflammatory cues [46, 76]	Immune–angiogenic crosstalk [27]

*TLR* Toll-like receptor, *PRR* pattern-recognition receptor, *ISG* interferon-stimulated gene, *IFN* interferon, *MMP* matrix metalloproteinase, *PJI* periprosthetic joint infection, *LPS* lipopolysaccharide, *IL* interleukin, *TNFα* tumour necrosis factorα, *CXCL and CCL* C-X-C and C-C motif chemokines, *VEGF* vascular endothelial growth factor

Several studies linked these responses to pathogen-specific response programmes. *Staphylococcus aureus* infection typically elicited robust induction of CXCL8 and CCL2, which drive neutrophil and monocyte recruitment, respectively, and was frequently accompanied by matrix metalloproteinase (MMP) activation that promotes extracellular matrix (ECM) degradation and tissue remodelling, whereas *Mycobacterium tuberculosis* exposure induced a more interferon-driven, CXCL10-dominated profile associated with T cell recruitment and activation [38]. Although studies specifically focusing on osteocytes remain limited, available in situ analyses of osteomyelitic and PJI bone demonstrate that osteocytes exhibit detectable cytokine and chemokine expression, indicating their capacity to actively participate in local inflammatory signalling and immune cell recruitment in response to infection [20, 29]. In these settings, osteocytes expressed inflammatory mediators and matrix-remodelling MMPs consistent with a paracrine role in immune cell recruitment and local tissue restructuring.

### Acquisition of APC-like Machinery in Osteoblasts

Several high-quality studies demonstrated that osteoblasts can process and present antigenic peptides to CD4⁺ T cells and directly contribute to T cell activation (Table 3) [11, 12, 39]. Under basal conditions, osteoblasts were reported typically to express the more ubiquitous MHC class I at low levels and exhibit minimal or undetectable MHC class II expression. However, bacterial infection, pro-inflammatory cytokines (e.g. IFN-γ) and innate immune ligands induced robust upregulation of MHC class II transcripts and protein in human and murine primary osteoblasts, and in osteoblast-like cell lines, including MG-63, Saos-2 and MC3T3-E1 [11, 12, 39, 40]. Importantly, this induction was accompanied by increased expression of CD74 (invariant chain) [41], which associates with newly synthesised MHC class II molecules and directs their trafficking to endosomal compartments, where it is proteolytically cleaved to form CLIP, which together prevent premature antigenic peptide binding. This was followed by increased expression of HLA-DM, a peptide-editing chaperone that removes CLIP and facilitates loading of high-affinity antigenic peptides. This process enables surface expression of peptide–MHC class II complexes for antigen presentation. Concurrent upregulation of lysosomal proteases, including cathepsins, and endosomal trafficking machinery further supports antigen processing and efficient presentation to CD4⁺ T cells [42–44].

**Table 3:** Evidence for APC-like machinery in osteoblasts

APC Feature	Evidence in Osteoblasts	Inducing Conditions	Functional Outcome
MHC class II	Upregulated in infected mouse [11] and human OB [11, 12]	*S. aureus*, *S. enterica*, IFN-γ, Poly I:C [11, 12]	Increased cell surface expression [11, 12]
HLA-DM	Present in infected OB	Infection, inflammation	Peptide editing capability
Cathepsins	Induced by pathogens	Endosomal trafficking	Supports antigen processing
Co-stimulation	CD40, CD80, CD86 [11, 12, 23, 39, 40, 49]	PRR ligands [11]	Enhances T cell activation [11, 12, 39, 40]
Antigen presentation	Mouse osteoblast induced transgenic OVA-specific CD4^+^ T cell proliferation	*S. aureus* or *S. enterica* exposure then pulsed with OVA	Functional APC behaviour [12]

*APC* antigen-presenting cell, *CD* cluster of differentiation, *HLA-DM* human leukocyte antigen DM, *IFN-γ* interferon gamma, *MHC* major histocompatibility complex, *MLO-Y4* murine long bone osteocyte cell line, *OB* osteoblast, *OVA* ovalbumin, *PRR* pattern-recognition receptor, *Poly I:C* polyinosinic–polycytidylic acid, *S. aureus* Staphylococcus aureus, *S. enterica* Salmonella enterica

In both human primary and cell line models, osteoblasts upregulated co-stimulatory molecules, including CD40 and, to a lesser extent, CD80 and CD86, following antigenic stimulation. This response was observed in models using live bacterial infection, particularly *S. aureus*, as well as defined PRR agonists such as lipopolysaccharide (LPS; stimulating TLR4) and poly I:C (stimulating TLR3), indicating activation of innate immune signalling pathways [11, 39, 40]. Functional co-culture experiments demonstrated that activated osteoblasts induced activation and proliferation of CD4⁺ T cells, as evidenced by upregulation of activation markers (e.g. CD69, CD25) and cytokine production [12].

### Osteocyte-Specific Immune Signatures in Deep Bone

**Fig. 2:**
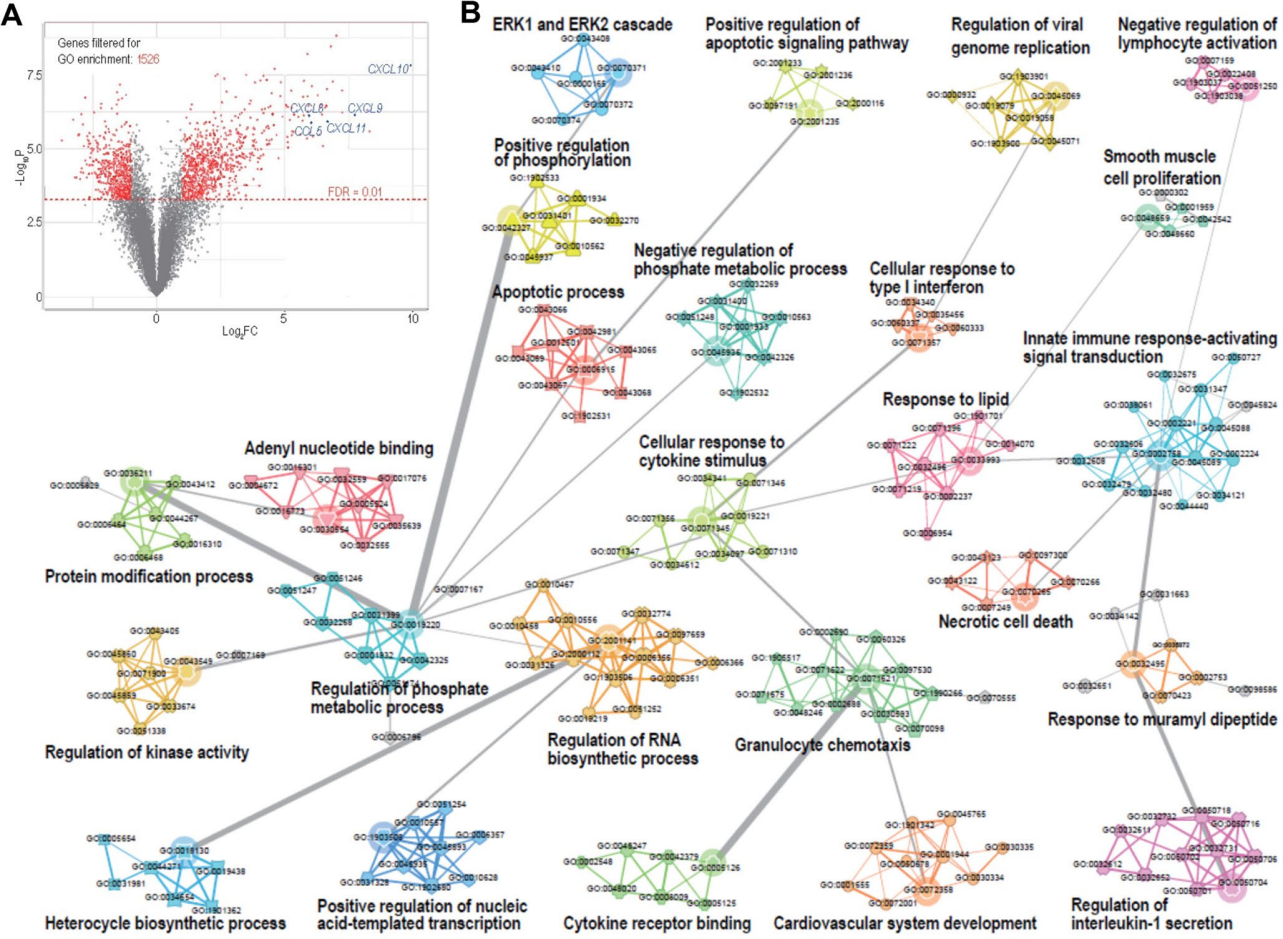
Human primary osteocyte response to bacterial infection Bioinformatics analysis of microarray data for human primary osteocytes exposed for 24 h to *S. aureus*. (A) Volcano plot showing differentially expressed (DE) genes and cut-off values for inclusion in the gene ontology (GO) enrichment analysis (x-axis represents the Log_2_ values of mRNA fold changes (FC) and y-axis represents the Log_10_*p* values of significance). (B) Enriched GO terms formed into communities based on the proportions of common DE genes between each pair of nodes. Each node represents a single GO term, with edges denoting the degree of connectedness between terms. Thicker edges correspond to a higher proportion of shared DE genes. Community hubs were defined as those with the most connections within each community and were used as community labels. The network layout and edge lengths were generated using a variation on the force directed model. (Reproduced under Creative Commons license CC-BY-4.0 from Yang D and Wijenayaka AR, et al.. Novel Insights into Staphylococcus aureus Deep Bone Infections: the Involvement of Osteocytes. Mbio. 2018;9(2):10.1128/mbio. 00415 − 18. doi: 10.1128/mBio.00415-18])

As summarised in Table 4, in infected or inflamed bone, osteocytes were reported to exhibit upregulation of PRR-associated transcripts, inflammatory mediators and matrix-remodelling enzymes, frequently in spatial association with bacterial invasion of the lacunocanalicular network [28, 29]. In a microarray-based study, Yang and Wijenayaka et al. identified a coordinated and extensive response by human primary osteocyte-like cells to acute *S. aureus* exposure. While they confirmed the transcription and release of CXCL8 (neutrophil response) and CCL5 and CXCL10 (T cell response), the overall response involved the significant differential expression of more than 1,500 genes, which could be assigned to one or more immune pathways, including innate immune response activating signal transduction, cellular response to type I interferon, regulation of IL-1 secretion, and negative regulation of lymphocyte activation pathways, among many others (Fig. 2). Transcriptomic analysis of both experimentally-infected human bone and bone taken from PJI patients recapitulated the findings in cultured osteocytes , thus extending the concept of innate and potentially adaptive immune sensing into the mineralised compartment.

Although direct evidence for antigen presentation by osteocytes remains limited, several studies reported induction of antigen-processing components and cathepsin-rich lysosomal compartments in osteocyte-like cells or osteocytes in situ under inflammatory or infectious conditions [28, 29]. While further studies are required, the studies to date suggest that osteocytes are biochemically poised to participate in immune modulation and support a model, in which osteocytes function as deep-bone immune sentinels.

**Table 4:** Immune signatures identified in osteocytes

Immune category	Specific mediator	Pathological context	Functional role
Pattern-recognition signalling	PRR-associated transcripts; TLR-related gene expression [28]	Osteomyelitis, PJI [10, 20, 29]	Detection of microbial or inflammatory danger signals within the lacunocanalicular network [20, 28, 29]
Inflammatory mediators	IL-6, chemokines [10]	Infected bone [10]	Paracrine inflammatory signalling and modulation of the local immune microenvironment
Matrix remodelling	MMPs [20, 29]	*S. aureus* canalicular invasion [10, 20, 29]	Osteocytic osteolysis and local matrix remodelling [29]
Antigen processing	HLA-DM, cathepsins	Inflammatory or infectious bone conditions [10, 29]	Potential antigen handling
Extracellular signalling	Extracellular- associated immune mediators inferred	Mechanical strain, infection [10, 29]	Deep-bone communication [10, 20, 29]

*HLA-DM* human leukocyte antigen DM, *IL-6* interleukin-6, *MMP* matrix metalloproteinase,* PJI* periprosthetic joint infection, *PRR *pattern-recognition receptor, *TLR* Toll-like receptor

### Pathogen- and Context-Specific Immune Response Programmes

Immune behaviour across the osteoblast lineage appears not to be uniform; instead, pathogen identity and inflammatory context impart distinct and highly specialised response programmes (Table 5). In human osteomyelitic bone samples and experimental models of *S. aureus*–induced osteomyelitis and PJI, osteoblasts and osteocytes exhibited upregulation of defined neutrophil- and monocyte-recruiting chemokines, including CXCL8, CXCL1 and CCL2, alongside induction of antimicrobial peptides, such as β-defensins. These responses, closely linked to TLR2/4 signalling [28, 29], were accompanied by activation of matrix metalloproteinases (e.g. MMP-2 and MMP-9), promoting bone matrix degradation and tissue remodelling within infected bone [20, 29, 45]. This was also demonstrated in human PJI patient bone in response to a broad range of Gram-positive and Gram-negative bacterial pathogens [20]. This suggests coordinated activation of inflammatory signalling pathways to facilitate immune cell infiltration and structural adaptation of the infected bone.

**Table 5:** Pathogen-specific immune patterns across the osteoblast lineage

Pathogen/Stimulus	Osteoblast Response	Osteocyte response	Overall program
*S. aureus*	IL-6, CXCL8, CXCL10 CCL2, β-defensins, MMPs [23, 26, 38, 77, 78]	MMPs, inflammatory mediators [10, 28, 29]	Acute inflammation and osteolysis [10, 20, 29]
*M. Tuberculosis*	CXCL2, CXCL8, CXCL10, IFN programs [37, 40, 58]	ISG activation	Chronic granuloma formation [37, 40]
*Salmonella*	IL-6, IFN- γ, MHC class II, CD80, CD86 [11, 45]	Cytokine and chemokine production	Deep bone infection and immune response modulation [11, 18]
*P. gingivalis*	CCL2, CXCL5, CXCL10, IL-6, IL-17, MMP13, RANKL [73]	Cytokine and chemokine-mediated inflammation	Bone mineral release and matrix degradation [73]
Poly I:C	IFN-β, ISGs [26]	Likely cGAS–STING	Type I IFN response [26]

*APC* antigen-presenting cell, *CD* cluster of differentiation, *CCL* C–C motif chemokine ligand,* CXCL* C–X–C motif chemokine ligand,* IFN* interferon, *IFN-β* interferon beta,* IFN-γ* interferon gamma, *IL* interleukin, *ISG* interferon-stimulated gene,* MHC* class II major histocompatibility complex class II, *MMP* matrix metalloproteinase, *Poly I:C* polyinosinic–polycytidylic acid, *PRR* pattern-recognition receptor, *RANKL* receptor activator of nuclear factor κB ligand, S. aureus *Staphylococcus aureus*, M. tuberculosis *Mycobacterium tuberculosis*, P. gingivalis *Porphyromonas gingivalis*

In contrast, *Mycobacterium tuberculosis*–associated models exhibited a more type 1 interferon-skewed, CXCL10-enriched chemokine profile, accompanied by altered RANKL/OPG dynamics and immune signalling patterns that favour chronic granulomatous inflammation over acute neutrophil recruitment [40]. Viral mimetics, such as Poly I:C, as well as direct activation of the cGAS–STING pathway, induced yet another distinct programme, characterised by a type I interferon response; in osteoblast and osteocyte-like cell models these responses have also been associated with altered osteogenic activity, including dysregulated expression of the master osteogenic transcription factor RUNX2 and alkaline phosphatase, indicating a link between antiviral signalling and bone formation pathways [26, 34].

Biomaterial-modified implant surfaces, including titanium and titanium alloy implants with altered topography or surface coatings (e.g. roughened, hydroxyapatite-coated, or antimicrobial-modified), impose additional layers of immune modulation. In these implant-associated settings, osteoblast-lineage cells exhibited altered PRR expression and cytokine production, alongside changes in the coupling between osteogenic differentiation and immune signalling programmes [40]. Collectively, these findings highlight that osteoblast-lineage immune functions are not generic but suggest they are dynamically tailored to specific pathogens and microenvironmental contexts, shaping both the magnitude and chronicity of bone-associated inflammation.

### Osteoprogenitor Immune Functions

Human osteoprogenitor cultures undergoing osteogenic differentiation were shown to exhibit functional PRR signalling cytokine/chemokine secretion in response to inflammatory stimulation [38]. However, these cells displayed limited antigen-presenting capacity, characterised by low or absent expression of MHC class II and minimal upregulation of co-stimulators, such as CD80 and CD86. During maturation, osteoblasts emerged as immune-competent effector cells capable of mounting robust chemokine networks, producing antimicrobial peptides, and integrating PRR- and cGAS–STING–mediated pathogen sensing (Table 5).

## Discussion

### The Osteoblast Lineage as an Integrated Immune System

**Fig. 3:**
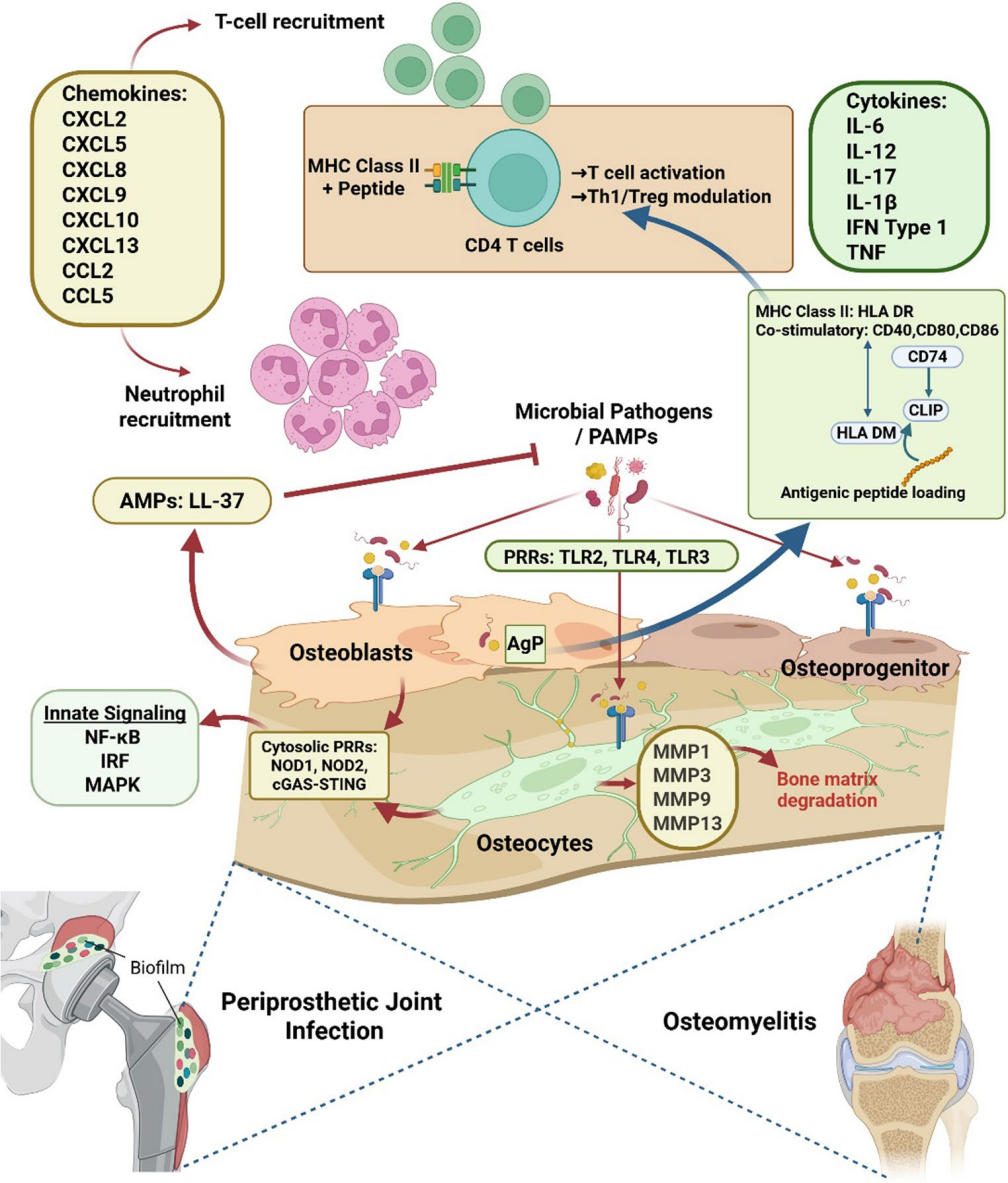
Evidence for the integrated immune functions of osteoblast-lineage cells in infected and inflamed bone Osteoprogenitors, osteoblasts, and osteocytes, respond to microbial and inflammatory stimuli such as microbial pathogens, TLR2/TLR4 ligands, IFN-γ, and the TLR3 ligand, the viral mimic Poly I:C. These stimuli activate innate immune sensing pathways involving pattern-recognition receptors (PRRs) including membrane-associated TLRs and cytosolic PRRs and downstream NF-κB, IRF and MAPK signalling. Osteoblasts and osteocytes release antimicrobial peptides (AMPs), such as LL-37, expected to directly impact the pathogen load. Both osteoblasts and osteocytes release proinflammatory chemokines involved in both neutrophil and T-cell recruitment. Osteocytes in particular release matrix metalloproteinases (MMPs) capable of degrading bone matrix. Both osteoblasts and osteocytes also release proinflammatory cytokines capable of immune cell proliferation and activation. In parallel, best characterised in osteoblasts, osteoblast-lineage cells acquire antigen-presenting (AgP) features, including expression of MHC class II (e.g. HLA-DR) and co-stimulatory molecules, such as CD40, CD80 and CD86. Components identified of the MHC class II pathway include expression of CD74 (invariant chain), which associates with newly formed MHC class II complexes, CLIP, which occupies the peptide-binding groove and the critical chaperone HLA-DM, which facilitates CLIP removal and antigenic peptide loading, enabling formation of peptide–MHC class II complexes for surface presentation. Collectively, these processes support communication with CD4⁺ T cells, promoting T-cell activation and modulation of Th_1_/T_reg_ responses within infected bone microenvironments, as present in osteomyelitis and periprosthetic joint infection settings. *Created in BioRender. Hossain*,* M*,* Atkins*,* G. (2026)* https://BioRender.com/nn461jl

The integration of evidence from the identified studies necessitates a fundamental reappraisal of the osteoblast lineage. Rather than serving solely as structural and metabolic custodians of skeletal integrity, the evidence suggests that osteoblast lineage cells together constitute a distributed, tissue-resident immune organ embedded within bone, a model we depict in Fig. 3. This framework reconciles previously fragmented observations of osteoblast-derived cytokines, osteocyte inflammatory signatures, antigen-processing machinery and bone immune interactions into a unified biological model **(**Tables 2 and 3) [11, 12, 29, 39, 46]. When considered along the osteogenic differentiation continuum, these findings suggest that immune functionality is distributed and specialised across all stages of osteoprogenitor, osteoblast and osteocyte. Within this lineage-integrated architecture, surface osteoblasts operate as dynamic immune effectors and conditional antigen processors [12], whereas osteocytes function as deep-matrix immune sentinels, sensing microbial invasion and inflammatory perturbations within the lacunocanalicular network [20]. This conceptual shift reframes this lineage not merely as structural or metabolic gatekeepers, but as an immunologically active tissue system, with implications extending across infectious disease, biomaterials integration, chronic inflammatory pathology and systemic immune regulation.

### The Osteoblast Immune Repertoire

The most compelling mechanistic insight emerging from this systematic review is that osteoblasts possess a far more sophisticated immune repertoire than is generally appreciated in the broader musculoskeletal field. Through expression of Toll-like receptors, NOD-like receptors and the cGAS–STING axis, osteoblasts are equipped to detect a broad spectrum of microbial and danger-associated signals [47, 48]. Rapid induction of IL-6, CXCL8, CCL2, CXCL10 and β-defensins positions these cells as frontline innate effectors in response to bacterial, viral and biomaterial-associated stimuli. Importantly, this response is not merely accessory, with multiple studies demonstrating that osteoblast activation directly influences the recruitment and functional programming of neutrophils, monocytes and T cells within infected or inflamed bone microenvironments (Table 1) [23, 34].

Strikingly, several studies demonstrated that osteoblasts can upregulate expression of MHC class II, the critical peptide chaperone and editing system CD74 and HLA-DM, lysosomal cathepsins, and key co-stimulatory molecules, including CD40, CD80, CD86, following stimulation by pathogens or inflammatory cytokines [11, 12, 39, 41, 49]. This inducible antigen-presenting phenotype establishes osteoblasts as conditional, non-professional APCs, creating a direct mechanistic bridge between innate sensing and adaptive immune activation. APC-like behaviour has been reported in non-professional stromal and epithelial cell types, however, the strategic localisation of osteoblasts at the bone–marrow interface places them in a unique position to influence antigen presentation to infiltrating and resident CD4⁺ T cells.

Collectively, these findings indicate that osteoblasts possess a spectrum of APC-like potential, ranging from innate antigen presenters with limited costimulatory capacity to contextually ‘upgraded’ effectors that approach quasi-professional functionality for adaptive immune responses during inflammation. A critical immunological aspect of their potential immunomodulatory function is reflected in their conditional surface phenotype: while infection robustly induces MHC class II and CD40 expression, the upregulation of classical co-stimulatory molecules, particularly CD80 and CD86, remains relatively limited. This comparatively low co-stimulatory capacity—relative to dendritic cells, which express high levels of CD80/CD86 required for full T-cell activation—suggests that osteoblasts may support partial T-cell activation or modulate T-cell responses rather than drive strong effector priming. In canonical adaptive immunity, antigen presentation in the absence of strong co-stimulation typically results in T-cell anergy rather than activation [50–52]. Therefore, we postulate that this differential expression may function as a context-dependent regulatory checkpoint that limits excessive T-cell activation when antigen-presenting pathways are induced. However, during active infection, the concomitant release of pro-inflammatory cytokines, such as IL-6 and IL-12, likely functions as a compensatory ‘third signal’, which can override the tolerogenic default [53]. Specifically, IL-6 is known to block the generation of tolerogenic T_reg_ cells and instead drive differentiation toward the inflammatory Th_17_ lineage [54, 55], while IL-12 can substitute for CD28 co-stimulation to support cytotoxic effector function [53]. Thus, the cytokine milieu effectively serves as a contextual switch, allowing osteoblasts to bypass the strict requirement for CD80/86 co-stimulation and actively engage adaptive immunity when pathogen danger is detected.

### Osteocytes: Deep-bone Immune Sentinels with Antigen-Processing Potential?

As the most prevalent and interconnected bone-resident cell type, osteocytes are an attractive candidate for sensing and responding to infection. However, their bony location suggests they are unlikely able to physically contact a passing T cell to form the traditional ‘immunological synapse’ as in lymphoid organs, required for antigen presentation [1, 20, 56]. Osteocytes retain extensive contact with surface osteoblasts via dendritic processes [1]. These processes have also been demonstrated to extend into the vascular facing surface and bone marrow in mouse calvarial bone [56], suggesting a potential means to directly contact T cells and other immune effector cells.

Osteocyte-enriched transcriptomic profiles (Fig. 3) and in situ histological analyses demonstrated that osteocytes are capable of mounting robust responses to infection by *S. aureus* and to chronic inflammatory signals (Table 5) [28, 29]. These responses included gene expression upregulation of PRR–associated and interferon-stimulated genes, consistent with activation of innate and possibly adaptive immune sensing pathways, as well as matrix-remodelling and inflammatory programmes. Specifically, osteocytes exhibited increased expression of inflammatory mediators, MMPs, cytokine-associated signalling components and chemokine-related transcripts and protein release, reflecting a coordinated response that couples immune activation with structural adaptation of the mineralised matrix [20, 29]. In addition, osteocytes from infected bone, notably from patients with PJI, display a marked bone matrix degradation response [20]. This may serve to both increase bone tissue fluid flow and increase access of the infected areas of bone to immune cells. Taken together, these observations position osteocytes as at least an embedded innate immune layer within bone, a population of deeply located cells capable of sensing, interpreting and transmitting inflammatory and microbial danger signals within the mineralised matrix. However, it is not yet known whether osteocytes participate directly in antigen presentation. 

### Pathogen- and Context-Specific Immune Programming Across the Lineage

Collectively, the pathogen- and context-specific immune programmes identified a model, in which immune functions of the osteoblast lineage are not ancillary but adaptive, discriminatory and dynamically tuned to microenvironmental challenge. *S. aureus* induces a distinct pyogenic programme characterised by CXCL8- and CCL2-driven neutrophil recruitment, β-defensin production and activation of osteolytic pathways, features consistent with its capacity for canalicular invasion and rapid expansion within bone [37, 57]. By contrast, *Mycobacterium tuberculosis* elicits a transcriptional landscape dominated by type 1 interferon signalling and CXCL10 release, mirroring the chronic, granulomatous nature of tuberculous osteomyelitis [40, 58]. Viral mimetics and nucleic acid-sensing ligands engage intracellular cGAS–STING pathways, driving interferon responses alongside upregulation of MHC class II and HLA-DM, thereby linking innate nucleic acid sensing to antigen-presentation capacity [34].

Beyond infectious stimuli, biomaterial interfaces can further modulate osteoblast lineage immune behaviour by reshaping cytokine output and osteogenic–immune coupling [38], demonstrating that osteoblast-lineage cells integrate microbial ligands, inflammatory mediators and biomaterial-derived signals to calibrate their immunological states [11]. It is possible that these immunological states are temporally regulated, with early responses dominated by innate sensing followed by later shifts toward antigen-presentation and the adaptive response, depending on stimulus or pathogen persistence and tissue context.

### Implications for bone Infection, Biomaterials and Systemic Immunity

The recognition of osteoblast-lineage cells as active participants in immune surveillance provides a mechanistic framework for several longstanding and unresolved clinical challenges. In chronic osteomyelitis, osteoblast and osteocyte immune responses may paradoxically contribute to bacterial persistence. Osteocyte infection by *S. aureus* enables microbial sequestration and persistence within bone tissue, seemingly beyond the effective reach of professional immune cells and systemic antibiotics [13, 29, 59, 60]. Simultaneously, sustained activation of osteoblast PRR pathways and cytokine circuits may generate a chronically inflamed microenvironment characterised by persistent proinflammatory mediator production, ineffective antimicrobial clearance and uncoupling of inflammation from pathogen eradication, thereby promoting bone destruction while failing to fully resolve the infection [20, 28, 29]. This duality offers a potential explanation for the high recurrence rates and therapeutic resistance observed in chronic bone infections.

In the context of biomaterials and orthopaedic implants, the findings indicate that osteoblast lineage cells act as first-line immune interpreters of implant-associated cues. For example, activation of osteoblast TLR2/TLR4 pathways by bacterial ligands or surface-associated stimuli induces robust IL-6, CXCL8, CCL2 and CXCL10 production, driving neutrophil and monocyte recruitment at the bone-implant interface (Tables 1 and 2). Conversely, differential engagement of PRR-linked signalling pathways and cytokine circuits modulates the balance between inflammatory signalling and osteogenic–immune coupling, influencing whether the peri-implant microenvironment evolves toward persistent inflammation or stable osseointegration. These observations support a paradigm of immuno-informed biomaterial design, in which implants are engineered not solely for mechanical integration but to shape osteoblast lineage immune outputs, such as limiting excessive pro-inflammatory cytokine and chemokine release, thereby biasing the local environment toward regenerative rather than inflammatory pathways. While not captured in the current review, in addition to the classical macrophage activation pathway, osteoblast lineage cells including progenitors, osteoblasts and osteocytes, have been shown to react profoundly to the particulates of biomaterials generated by implant wear, so called ‘particle disease’, associated with implant aseptic loosening [61–63]. The principal consequence of this process is osteoblastic activation and differentiation of bone resorbing osteoclasts through proinflammatory upregulation of RANKL, while osteocytic osteolysis has also been described in this context [61, 62]. How these responses involve and influence the classical innate and adaptive immune pathways requires further exploration.

Beyond local bone pathology, our synthesis suggests that osteoblast-lineage immune activity may exert systemic effects. Experimental models linking osteoblast dysfunction with altered haemopoietic support by altering key niche factors such as CXCL12, and inflammatory cytokines, leading to impaired lymphoid development, skewed myeloid output and features of immunodeficiency [31, 36]. In this context, osteoblast-lineage immune signalling has been shown to regulate leukocyte recruitment and to shape T-cell priming environments through sustained chemokine and cytokine production [12, 23, 30, 37]. Consequently, pathological alterations in bone immunity, such as that in chronic osteomyelitis, PJI and inflammatory bone diseases, characterised by prolonged PRR activation and cytokine release, are likely to have effects that extend beyond the skeleton, contributing to systemic immune imbalance and altered host defence [5, 31, 64].

### Future Directions and Unresolved Questions

Despite the emerging recognition of immune functions across the osteoblast lineage, several critical mechanistic questions remain unresolved. While osteoblasts have been shown to express key components of the antigen-processing machinery, it remains unknown whether osteoprogenitors or osteocytes can acquire similar antigen-presenting capacities under inflammatory or infectious conditions. Another major uncertainty concerns the downstream consequences of osteoblast antigen presentation; whether osteoblast-mediated signalling preferentially drives Th_1_, Th_17_ or T_reg_ cell differentiation likely depends on both pathogen type and inflammatory context, but this has not yet been systematically explored. Furthermore, while there are clear influences on B lymphopoiesis [8], the potential participation of osteoblast lineage cells in humoral responses requires elucidation. From a translational perspective, determining whether targeted modulation of osteoblast lineage immune pathways can improve outcomes in chronic osteomyelitis or PJI represents an important future therapeutic avenue.

The interplay between active infection of osteoblast lineage cells and their ability to mount a protective immune response also deserves further research. The wide range of pathogens reportedly able to persist in osteoblasts and osteocytes both in vitro and in vivo [13], could suggest that these cells are simply vulnerable to infection. However, in most cases, this is in the context of chronic infections (e.g. chronic PJI, periodontitis, Chlamydial bone and joint inflammation, musculoskeletal tuberculosis), so active osteoblastic infection may be more reflective of disease severity and/or be mechanistically linked to the respective chronic disease state. Conversely, where exposure of the bone to infective agents does not result in chronic osteomyelitis or inflammation may be reflective of an efficient osteoblastic immune response.

An obvious area of further research is detailing how the immune functions of osteoblast lineage cells during an active infection interact with the ‘classical’ osteoimmunology and bone remodelling pathways. Osteomyelitis involves increased bone resorption by osteoclasts, osteocyte-mediated bone matrix degradation, as well as new bone formation, including the involucrum surrounding necrotic, infected areas (sequestra) and periosteal apposition [20, 64]. Dissecting pathogen and host osteoblastic immune effects on these processes will be of significant interest. Finally, the integration of PRR activation, interferon signalling and potential APC-like functions during infection or inflammation with mechanotransduction pathways in osteoblast lineage cells remains an unexplored frontier. Resolving these questions will require interdisciplinary approaches that combine skeletal biology, immunology, systems biology and high-resolution imaging.

## Conclusions

This systematic review demonstrates that immune function is not an incidental property of the osteoblast lineage, but rather an intrinsic feature distributed along the osteogenic differentiation continuum. Rather than transitioning from immune-competent progenitors to immune-silent matrix embedded cells, the evidence suggests that osteogenic maturation involves the spatial and functional specialisation of immune capabilities. Mature osteoblasts emerge as conditional antigen-presenting cells integrating PRR signalling, cytokine production and MHC class II–dependent antigen processing, while osteocytes exhibit signatures of innate and to some extent adaptive immune activation in infected and inflamed bone, consistent with the role of serving as deep-matrix immune sentinels.

Together, these findings redefine osteoblast-lineage cells as an immune tissue composed of a coordinated osteoblast–osteocyte network that senses microbial danger, shapes leukocyte recruitment, modulates adaptive immune activation and communicates immunological information through the lacunocanalicular system. This lineage-integrated immune architecture reframes the pathobiology of osteomyelitis, biomaterial integration and inflammatory bone loss, and establishes a conceptual and mechanistic foundation for future therapeutic strategies targeting osteoblast lineage–immune crosstalk in skeletal and potentially systemic disease.

## Key References

- Schrum LW, Bost KL, Hudson MC, Marriott I. Bacterial infection induces expression of functional MHC class II molecules in murine and human osteoblasts. Bone 2003; 33: 812-821 [12]expression of functional MHC class II molecules in murine and humanosteoblasts. Bone 2003; 33: 812-821 [12]
  - This landmark study demonstrated that bacterial pathogens *S. aureus* and Salmonella induce robust expression of functional MHC class II molecules in bothmurine and human osteoblasts, mediated in part by upregulation of the transcriptional regulator CIITA.
- Ormsby RT, Zelmer AR, Yang D, Gunn NJ, Starczak Y, Kidd SP, et al. Evidence for osteocyte-mediated bone-matrix degradation associated with periprosthetic joint infection (PJI). Eur Cell Mater. 2021;42:264-80. doi:10.22203/eCM.v042a19 [20].
  - This study showing that the osteocyte elicits a bone matrix degradation response, with evidence this is MMP-mediated, to a wide range of pathogens in human cases of PJI suggests an apparent new facet of the host immune response in osteomyelitis.
- Grcevic D, Sanjay A, Lorenzo J. Interactions of B-lymphocytes and bone cells in health and disease. Bone. 2023;168:116296. doi:10.1016/j.bone.2021.116296 [8].
  - This excellent review presents a detailed account of the reciprocal interactions between B cells and osteoblast lineage cells, in terms of osteoimmunological effects of B cells on bone remodelling and the roles of osteoblastic cells in B lymphopoiesis.

## Data Availability

No datasets were generated or analysed during the current study.
